# Supplementary material for: Cerebellar climbing fibers multiplex movement and reward signals during a voluntary movement task in mice
Source: Commun Biol. 2023 Sep 9;6:924. doi: 10.1038/s42003-023-05309-9 (PMC10492837; doi:10.1038/s42003-023-05309-9)
Supplement: Supplementary file 2 — Supplemental Information [file 42003_2023_5309_MOESM2_ESM.pdf]

Supplementary Information

Cerebellar climbing fibers multiplex movement and reward signals during a voluntary movement task in mice

Koji Ikezoe, Naoki Hidaka, Satoshi Manita, Masayoshi Murakami, Shinichiro Tsutsumi, Yoshikazu Isomura, Masanobu Kano, Kazuo Kitamura

Correspondence:
Koji Ikezoe (kikezoe@yamanashi.ac.jp)
Masanobu Kano (mkano-tky@m.u-tokyo.ac.jp)
Kazuo Kitamura (kitamurak@yamanashi.ac.jp)

This PDF file includes:

Supplementary Figures 1 to 4

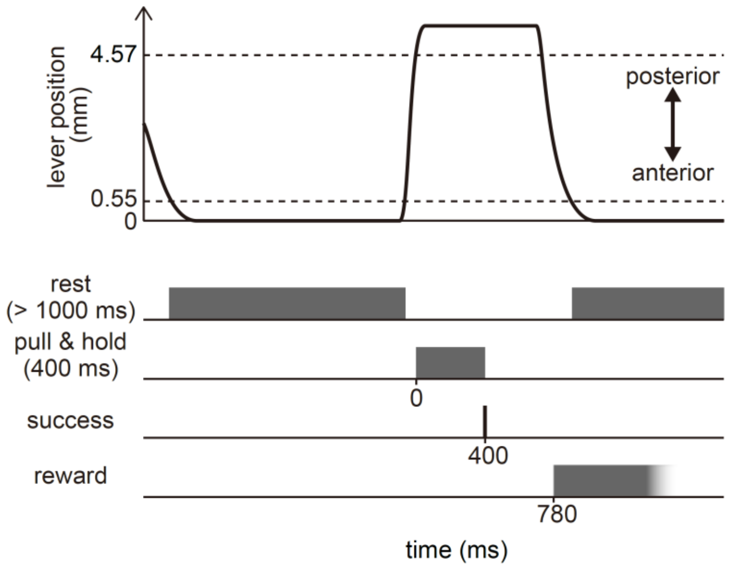

Supplementary Figure 1 | Timings of criteria in the lever pull task.
To obtain the water reward, mice have to keep the lever at the resting position (< 0.55 mm) for more than 1000 ms and then pull and maintain it beyond the higher threshold (> 4.57 mm) for 400 ms. Reward (4–8 μL water) is delivered 380 ms following a task success.

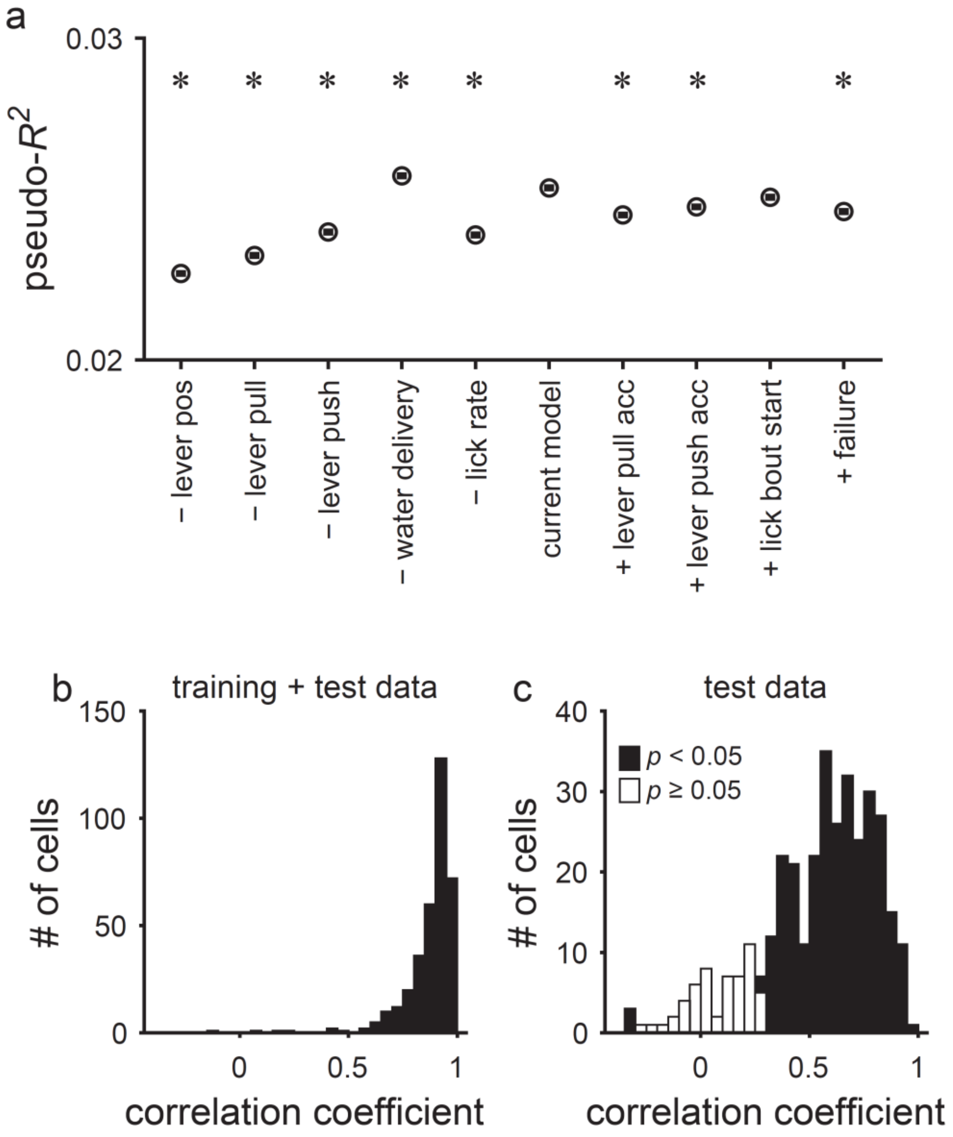

Supplementary Figure 2 | Prediction performance of the encoding model.
a Selection of the behavioral variables in the encoding model. Mean prediction performances (pseudo-R^2), explained deviance, of encoding models with different behavioral variables. A minus sign and a plus sign mean excluding and adding the parameter from and to the current model, respectively. Acc represents acceleration. Error bars: s.e.m. *: $p < 0.05$, two-sided Wilcoxon's signed-rank test. **b**, **c** Pearson's correlation coefficient between model prediction and PETH aligned to the start timing of lever pull in training and test data **b** and test data **c**, respectively.

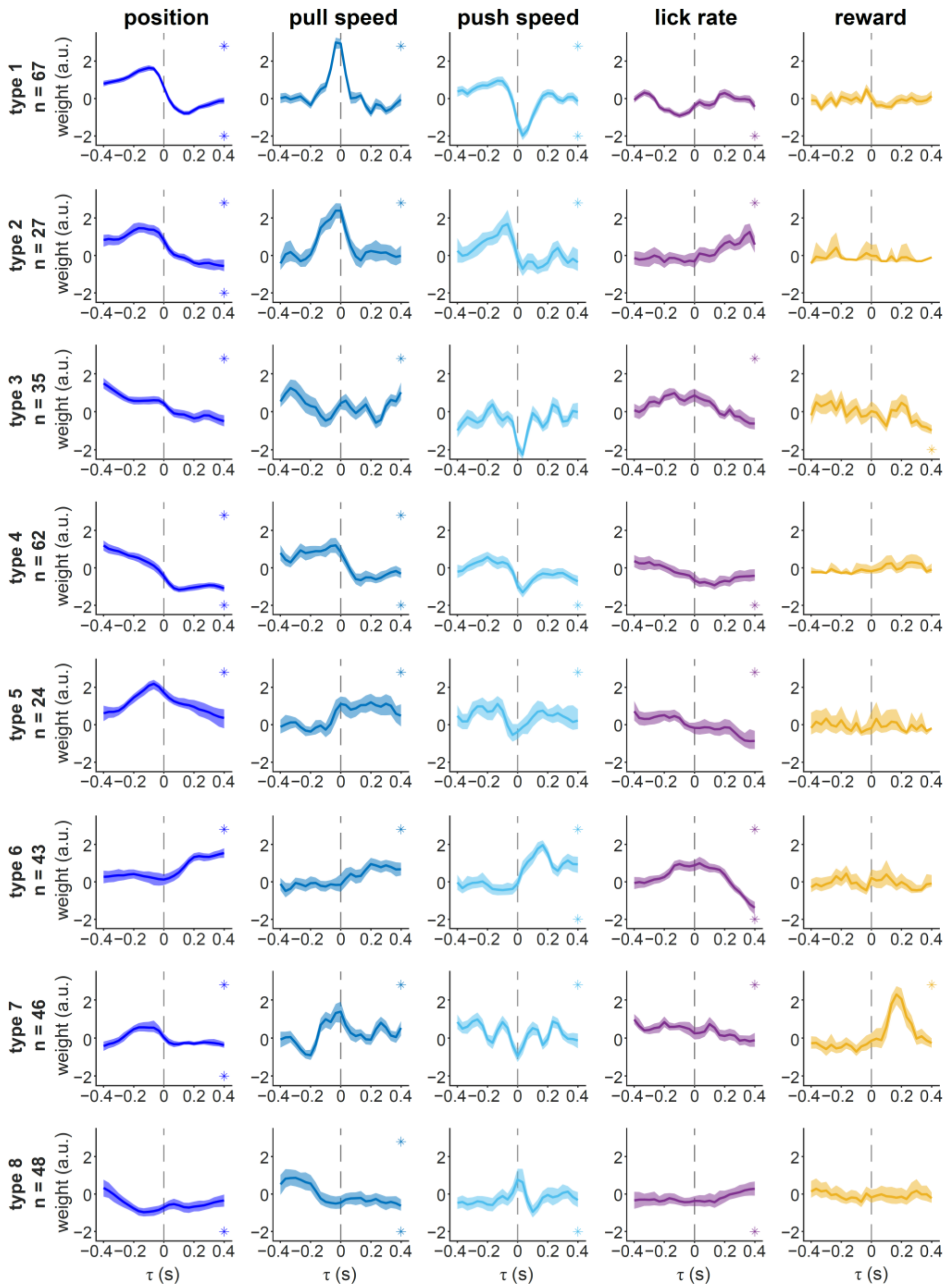

Supplementary Figure 3 | Individual plots of the response kernels shown in Figure 3b. Row: functional type of PCs. **Column:** behavioral variables. The asterisks in the first and fourth quadrant of each panel indicate that the mean of a variable significantly deviates from zero toward the positive and negative direction, respectively. Shade: 99% bootstrapped confidence interval of the mean.

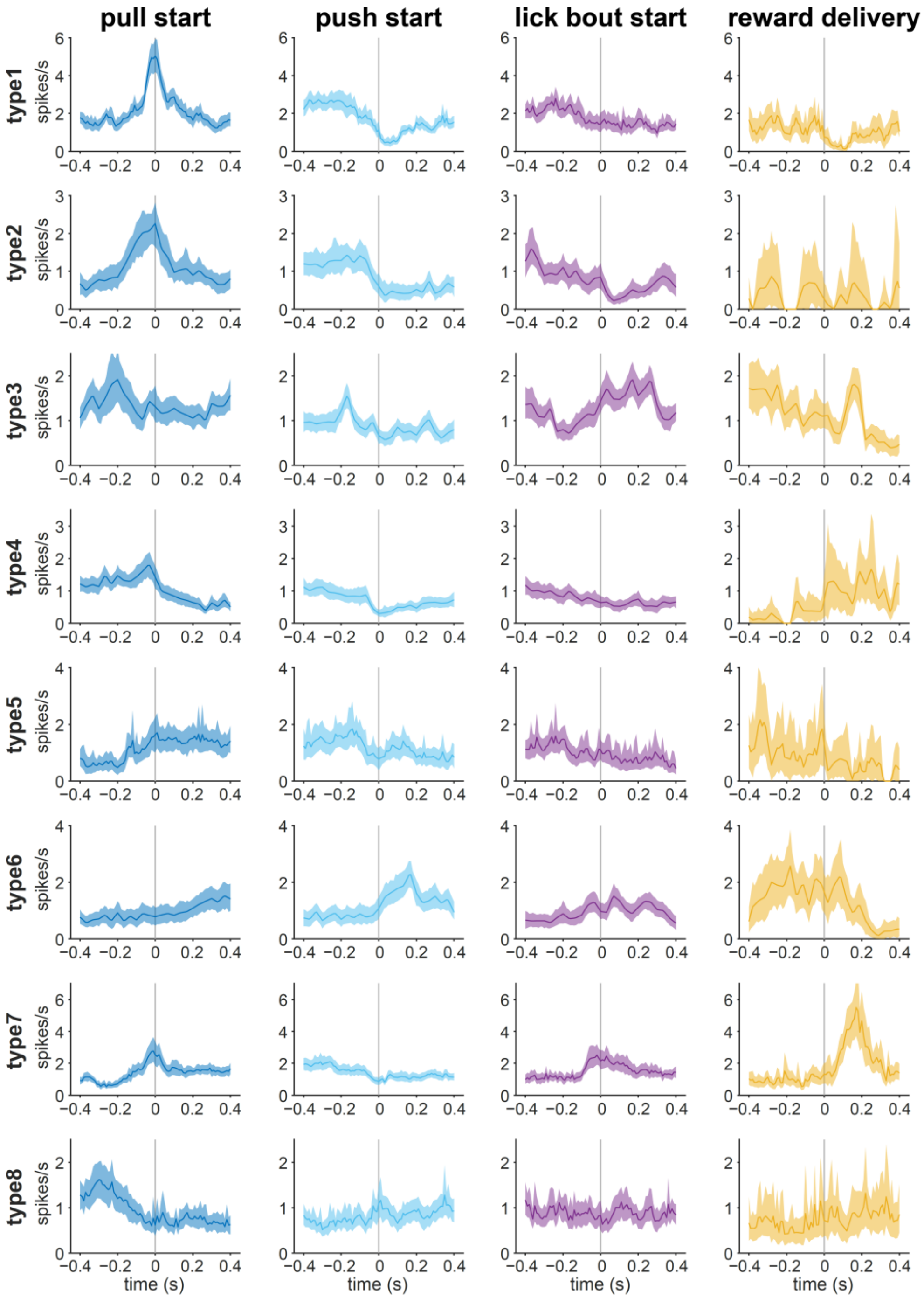

Supplementary Figure 4 | Population PETHs of dCSs of PCs in each functional type. Individual plots show the means of PETHs of PCs in the same functional types to one of the behavioral variables. **Row:** functional type of PCs. **Column:** Event types related to the behavioral variables for the encoding analysis. Shade: 99% bootstrapped confidence interval of the mean.
