# Supplementary material for: Cerebellar climbing fibers multiplex movement and reward signals during a voluntary movement task in mice
Source: Commun Biol. 2023 Sep 9;6:924. doi: 10.1038/s42003-023-05309-9 (PMC10492837; doi:10.1038/s42003-023-05309-9)
Supplement: Supplementary file 5 — Reporting Summary [file 42003_2023_5309_MOESM5_ESM.pdf]

nature portfolio

Corresponding author(s): Koji Ikezoe
Masanobu Kano
Kazuo Kitamura

Last updated by author(s): Aug 16, 2023

Reporting Summary

Nature Portfolio wishes to improve the reproducibility of the work that we publish. This form provides structure for consistency and transparency in reporting. For further information on Nature Portfolio policies, see our Editorial Policies and the Editorial Policy Checklist.

Statistics

For all statistical analyses, confirm that the following items are present in the figure legend, table legend, main text, or Methods section.

n/a	Confirmed	
☐	☒	The exact sample size (*n*) for each experimental group/condition, given as a discrete number and unit of measurement
☐	☒	A statement on whether measurements were taken from distinct samples or whether the same sample was measured repeatedly
☐	☒	The statistical test(s) used AND whether they are one- or two-sided<br>*Only common tests should be described solely by name; describe more complex techniques in the Methods section.*
☐	☒	A description of all covariates tested
☐	☒	A description of any assumptions or corrections, such as tests of normality and adjustment for multiple comparisons
☐	☒	A full description of the statistical parameters including central tendency (e.g. means) or other basic estimates (e.g. regression coefficient) AND variation (e.g. standard deviation) or associated estimates of uncertainty (e.g. confidence intervals)
☐	☒	For null hypothesis testing, the test statistic (e.g. *F*, *t*, *r*) with confidence intervals, effect sizes, degrees of freedom and *P* value noted<br>*Give P values as exact values whenever suitable.*
☒	☐	For Bayesian analysis, information on the choice of priors and Markov chain Monte Carlo settings
☒	☐	For hierarchical and complex designs, identification of the appropriate level for tests and full reporting of outcomes
☐	☒	Estimates of effect sizes (e.g. Cohen's *d*, Pearson's *r*), indicating how they were calculated

Our web collection on statistics for biologists contains articles on many of the points above.

Software and code

Policy information about availability of computer code

Data collection	Calcium imaging data was collected using ScanImage (Vidrio technologies). Behaivioral data was collected using a custom program written in LabView (National Instruments).
Data analysis	We performed data analysis using a custom program written in MATLAB (R2022a, MathWorks) and available softwares. Segmentation of calcium imaging data was performed using Suite2P(github.com/MouseLand/suite2p). Spike detection was performed using a custom program utilizing OASIS (github.com/j-friedrich/OASIS).

For manuscripts utilizing custom algorithms or software that are central to the research but not yet described in published literature, software must be made available to editors and reviewers. We strongly encourage code deposition in a community repository (e.g. GitHub). See the Nature Portfolio guidelines for submitting code & software for further information.

Data

Policy information about availability of data

All manuscripts must include a data availability statement. This statement should provide the following information, where applicable:

- Accession codes, unique identifiers, or web links for publicly available datasets
- A description of any restrictions on data availability
- For clinical datasets or third party data, please ensure that the statement adheres to our policy

The datasets generated during and/or analyzed during the current study are available from the corresponding author upon reasonable request.

Research involving human participants, their data, or biological material

Policy information about studies with human participants or human data. See also policy information about sex, gender (identity/presentation), and sexual orientation and race, ethnicity and racism.

Reporting on sex and gender	N/A
Reporting on race, ethnicity, or other socially relevant groupings	N/A
Population characteristics	N/A
Recruitment	N/A
Ethics oversight	N/A

Note that full information on the approval of the study protocol must also be provided in the manuscript.

Field-specific reporting

Please select the one below that is the best fit for your research. If you are not sure, read the appropriate sections before making your selection.

☒ Life sciences ☐ Behavioural & social sciences ☐ Ecological, evolutionary & environmental sciences

For a reference copy of the document with all sections, see nature.com/documents/nr-reporting-summary-flat.pdf

Life sciences study design

All studies must disclose on these points even when the disclosure is negative.

Sample size	No statistical methods were used to pre-determine sample sizes.
Data exclusions	Some mice were excluded from the analysis, because they did not learn the task, data could not be recorded from them due to poor expression of GCamp6f, or a sufficient number of ROIs were not detected by Suite2P due to low signal-to-noise ratio. ROIs whose activities were not predicted by the encoding model ($p > 0.05$) were excluded from the analysis of their responses (see Main text).
Replication	Cells showing a certain type of response properties (funcitonal type) were common in some imaging sites from different mice. Spatial clustering of cells according to their response properties were observed in most of imaging sites(Fig. 4, 5, and 6). Higher noise correlations between cells belonging to the same functional types than to different functional types were observed more than a half of imaging sites (Fig. 6d). Therefore, the main findings were replicated across mice.
Randomization	No randomization of mice was performed as there was only one experimental group.
Blinding	Data collection and analysis were not performed blind to the experiment, because data collection was performed in the same condition except GCamp6f expression procedure and analysis relied on code that was standardized across mice.

Reporting for specific materials, systems and methods

We require information from authors about some types of materials, experimental systems and methods used in many studies. Here, indicate whether each material, system or method listed is relevant to your study. If you are not sure if a list item applies to your research, read the appropriate section before selecting a response.

Materials & experimental systems

n/a	Involved in the study
☒	☐ Antibodies
☒	☐ Eukaryotic cell lines
☒	☐ Palaeontology and archaeology
☐	☒ Animals and other organisms
☒	☐ Clinical data
☒	☐ Dual use research of concern
☒	☐ Plants

Methods

n/a	Involved in the study
☒	☐ ChIP-seq
☒	☐ Flow cytometry
☒	☐ MRI-based neuroimaging

Animals and other research organisms

Policy information about studies involving animals; ARRIVE guidelines recommended for reporting animal research, and Sex and Gender in Research

Laboratory animals	We used C57BL/6J wild-type (WT) (C57BL/6JJmsSlc, Japan SLC, RRID:MGI:5488963) and PCP2 (L7)-Cre mice (Tg(Pcp2-cre)2Mpin/J, The Jackson Laboratory, RRID:MGI:3531210). We used adult (> 3 weeks) male mice.
Wild animals	No wild animals were used in this study.
Reporting on sex	We used male mice.
Field-collected samples	No field-collected samples were used in this study.
Ethics oversight	All experiments and animal protocols were approved by the Animal Experiment Committees of The University of Tokyo and University of Yamanashi.

Note that full information on the approval of the study protocol must also be provided in the manuscript.
